# Experimental investigation on the effects of website aesthetics on user performance in different virtual tasks

**DOI:** 10.7717/peerj.6516

**Published:** 2019-02-22

**Authors:** Meinald T. Thielsch, Russell Haines, Leonie Flacke

**Affiliations:** 1 Department of Psychology, University of Münster, Münster, Germany; 2 Department of Information Technology and Decision Sciences, Old Dominion University, Norfolk, VA, United States of America

**Keywords:** Transfer tasks, Search tasks, Creative tasks, Goal orientation, Digital work, Interface aesthetics

## Abstract

In Human-Computer Interaction research, the positive effect of aesthetics on users’ subjective impressions and reactions is well-accepted. However, results regarding the influence of interface aesthetics on a user’s individual performance as an objective outcome are very mixed, yet of urgent interest due to the proceeding of digitalization. In this web-based experiment (*N* = 331), the effect of interface aesthetics on individual performance considering three different types of tasks (search, creative, and transfer tasks) is investigated. The tasks were presented on an either aesthetic or unaesthetic website, which differed significantly in subjective aesthetics. Goal orientation (learning versus performance goals) was included as a possible moderator variable, which was manipulated by using different task instructions. Both aesthetics and goal orientation were a between-subject factor, leading to a 2 × 2 between subject design. Manipulation checks were highly significant. Yet the results show neither significant main effects of aesthetics and goal orientation on performance regarding both accuracy and response times in each of the three tasks, nor significant interaction effects. Nevertheless, from a practical perspective aesthetics still should be considered due to its positive effects on subjective perceptions of users, even as no substantial effects on user performance occurred in the present experiment.

## Introduction

Nowadays, digitalization is one of the most important and challenging topics in the modern working world. Organizations have to face the fact that virtual work is on the rise (Bell & Kozlowski, 2002; Hertel et al., 2017; Townsend, De Marie & Hendrickson, 1998). Many employees have to handle technical artifacts at work, particularly office workers who communicate, solve problems, or create ideas e.g., on a computer, mobile phone or tablet. Furthermore, the e-learning sector is growing: for example, the Silicon Valley based online university Udacity (https://de.udacity.com/) was founded as a start-up in 2012, but has soon become a matter of interest for several big organizations. Hence, proper design of software and web interfaces is of increasing importance in different contexts, be it for performance-oriented organizations or learning-oriented digital tools. Comparing the first-ever website (http://info.cern.ch/hypertext/WWW/TheProject.html) to a modern one, a huge change and advancement in design becomes obvious. Aesthetics in particular has gained a lot of attention in past years, since the positive effect of interface aesthetics on subjective impressions and reactions has been shown (e.g., Bargas-Avila & Hornbæk, 2011; Hassenzahl & Tractinsky, 2006; Thielsch, Blotenberg & Jaron, 2014; also see Moshagen & Thielsch, 2010, for an overview). For organizations, these subjective outcomes should be already interesting; however, the effect of aesthetics on objective outcomes, such as performance, might be of even more importance to achieve business success. Thus, the question arises whether an aesthetic interface is able to influence performance, which is investigated in the present experiment. In addition, some studies suggest that aesthetics effects might occur in particular in learning scenarios (e.g., Miller, 2011; Plass et al., 2014; Szabo & Kanuka, 1998). Pursuing learning goals means focusing on skill acquisition (Locke & Latham, 2006; Seijts & Latham, 2005) and comes along with positively connoted outcomes, such as feedback-seeking as well as task and job performance (Payne, Youngcourt & Beaubien, 2007). In contrast, performance goals underline the final result of a task with which individuals try to prove their competence (Elliott & Dweck, 1988) and are thus triggering extrinsic motivation. Unlike learning goals (Benware & Deci, 1984), performance goals might undermine intrinsic motivation (Dweck, 1986; Rawsthorne & Elliot, 1999). Thus, goal orientation (learning versus performance goals) was manipulated to test for influences of this variable.

### Aesthetics in human–computer interaction research

For a long time, research on human–computer interaction (HCI) has concentrated on usability (e.g., Hassenzahl & Tractinsky, 2006; Hornbæk, 2006), which is defined as ‘the extent to which a product can be used by specified users to achieve specified goals with effectiveness, efficiency and satisfaction in a specified context of use’ (ISO, 1998, p. 2). Aesthetics, in the past often seen just as a ‘nice-to-have’ facet, has gained attention in recent years due to many studies highlighting its potential (e.g., Bargas-Avila & Hornbæk, 2011; Hassenzahl & Tractinsky, 2006; Thielsch, Blotenberg & Jaron, 2014). Thus, aesthetics is no longer disregarded in HCI research and has evolved into a ‘must-have’ facet (Thielsch, Blotenberg & Jaron, 2014).

Psychological research on aesthetic perception dates back to the very first beginnings of experimental psychology (see Fechner, 1871; Wundt, 1874). Today, aesthetics is known as a multi-dimensional construct (Lavie & Tractinsky, 2004; Leder & Nadal, 2014; Moshagen & Thielsch, 2010), yet lacking a ubiquitous definition (see Thielsch, Blotenberg & Jaron, 2014). There are different tendencies in HCI research in defining aesthetics. For instance, Lavie & Tractinsky (2004) differentiate between ‘classical’ and ‘expressive’ aesthetics: ‘Classical’ aesthetics includes attributes like ‘clean’, ‘pleasant’ and ‘symmetrical’, whereas ‘expressive’ aesthetics is meant to be ‘sophisticated’ and ‘creative’. As another approach, Moshagen & Thielsch (2010) specify aesthetics to be an ‘immediate pleasurable subjective experience that is directed toward an object and not mediated by intervening reasoning’ (Moshagen & Thielsch, 2010, p. 690), which is the interactionist viewpoint we will draw on in the following.

Aesthetics is the main factor influencing the first and overall impression of a website, although people generally declare the content to have that role when giving a self-report (Thielsch, Blotenberg & Jaron, 2014). Especially in the recent years, many studies have emerged investigating not only subjective effects of aesthetics (e.g., on first impression, overall impression, intentional outcomes; Moshagen & Thielsch, 2010; Thielsch, Blotenberg & Jaron, 2014; Tuch et al., 2012), but also the rather objective effect of aesthetics on performance (e.g., Moshagen, Musch & Göritz, 2009; Reppa & McDougall, 2015; Sonderegger & Sauer, 2010).

### Current research on aesthetics and individual performance

There are several studies investigating the relationship between aesthetics and performance (for an overview see Thielsch & Niesenhaus, 2017). However, results regarding the influence of aesthetics on users’ performance as an objective outcome are very mixed; several theories are discussed, but no model fully explains the observed findings. Most of the studies on the topic are based on students’ samples and explaining models are often discussed post-hoc, whereas field studies, theory-driven experiments, systematic overviews or meta-analyses are lacking (Thielsch & Niesenhaus, 2017). Thus, this study is a theory-driven experiment using a sample from a more general population.

Multiple studies suggest that an aesthetic interface could enhance performance (e.g., Douneva, Jaron & Thielsch, 2016; Miller, 2011; Pomales-Garcia, Liu & Mendez, 2005; Sonderegger & Sauer, 2010; Strebe, 2016; Tuch et al., 2009; Um et al., 2012; Van Schaik & Ling, 2008), or that aesthetics has an at least partially positive effect on performance (e.g., Moshagen, Musch & Göritz, 2009; Plass et al., 2014; Reppa & McDougall, 2015). There are two prominent theories that try to explain a positive effect of aesthetics on performance: (1) the ‘positive affect mediation’ hypothesis (Norman, 2002; Norman, 2004), a cognitive theory, and (2) the ‘increased motivation’ hypothesis (Sonderegger & Sauer, 2010), a motivational theory. According to Norman (2002) and Norman (2004), an aesthetic interface might evoke positive emotions, which will in turn positively influence the cognitive system and thereby boost performance. In contrast, according to Sonderegger & Sauer (2010), an aesthetic interface may motivate and put users at ease (Lindgaard, 2007) and thus increases performance. More authors seem to post-hoc favor Norman’s theory (e.g., Moshagen, Musch & Göritz, 2009; Quinn & Tran, 2010; Reppa & McDougall, 2015).

On the other hand, some studies suggest a negative effect of aesthetics on performance (e.g., Sauer & Sonderegger, 2011; Sonderegger et al., 2014; Van Schaik & Ling, 2009). Such findings are in line with early concerns in the field of HCI that aesthetic designs interfere with work goals (e.g., Andre & Wickens, 1995; Hollnagel, 2003). One possible explanation for negative effects of aesthetics on performance is given by the ‘prolongation of joyful experience’ hypothesis (Sonderegger & Sauer, 2010). According to this line of reasoning, appealing interfaces lead to higher response times as people seek to extend the pleasurable experience, which therefore causes decrements in performance. As a further explanation for negative design effects, Gnambs, Appel & Batinic (2010) found that the color red is responsible for impeding men’s performance in web-based tests due to stereotype threats.

Hence, the current state of research is very equivocal, including studies showing a positive effect of aesthetics on performance, others showing a negative effect and, not to forget, many showing no effect at all (e.g., Ben-Bassat, Meyer & Tractinsky, 2006; Chawda et al., 2005; Douneva, Haines & Thielsch, 2015; Hall & Hanna, 2004; Ilmberger, Schrepp & Held, 2008; Katz, 2010; Ling & van Schaik, 2006; Sonderegger et al., 2012; Thüring & Mahlke, 2007).

Moreover, all those studies investigating the relationship between aesthetics and performance differ in many aspects. Firstly, the manipulation of aesthetics is diverse: for example, manipulations of just one facet (e.g., color; Hall & Hanna, 2004; Moshagen, Musch & Göritz, 2009) versus a broad manipulation (e.g., Miller, 2011; Sonderegger & Sauer, 2010). Secondly, the operationalization of performance varies, encompassing task duration (e.g., Chawda et al., 2005; Moshagen, Musch & Göritz, 2009), number of correct answers (e.g., Douneva, Jaron & Thielsch, 2016; Van Schaik & Ling, 2009), number of errors (e.g., Reppa & McDougall, 2015; Salimun et al., 2010), number of commands needed (e.g., Reinecke & Bernstein, 2011; Sonderegger & Sauer, 2010; Sonderegger et al., 2014), as well as comprehension and transfer (Plass et al., 2014; Um et al., 2012). Thirdly, the type of tasks differs, spanning classical search tasks (e.g., Tuch et al., 2009; Van Schaik & Ling, 2009), creative tasks (e.g.,  Bonnardel, Piolat & Le Bigot, 2011) or learning tasks (e.g., Heidig, Müller & Reichelt, 2015; Miller, 2011; Um et al., 2012). Fourth, the existing studies have been conducted with a variety of different products and interfaces for example ATM applications (e.g., Tractinsky, Katz & Ikar, 2000), Browser-based applications and websites (e.g., Douneva, Haines & Thielsch, 2015; Douneva, Jaron & Thielsch, 2016; Katz, 2010; Ling & van Schaik, 2006; Van Schaik & Ling, 2009), software (e.g., Reinecke & Bernstein, 2011), mobile phones (e.g., Quinn & Tran, 2010; Sauer & Sonderegger, 2011; Sonderegger & Sauer, 2010; Sonderegger et al., 2014), or portable digital audio players (e.g.,  Minge & Thüring, 2018; Thüring & Mahlke, 2007). Despite these many different starting-points, possibilities of manipulation, and perspectives, a systematic pattern of whether and how aesthetics impacts performance has not appeared yet and systematic research on proposed theories is lacking (Thielsch & Niesenhaus, 2017). In the present study, we aim to foster effects of aesthetics by using a broad manipulation of a website in different goal scenarios; asking participants to perform search, creative, and transfer tasks (see below) while using task duration and quality of answers as performance indicators.

### Goal orientation

The inconclusive evidence regarding aesthetics and performance implies to many that further variables play a role in this context. One likely variable is goal orientation (classified as learning versus performance goals), since most of the studies being located in a learning context show an at least partially positive effect of aesthetics on performance (Heidig, Müller & Reichelt, 2015; Miller, 2011; Plass et al., 2014; Pomales-Garcia, Liu & Mendez, 2005; Strebe, 2016; Szabo & Kanuka, 1998; Um et al., 2012), in contrast to several which are more performance-oriented that found no effect (e.g., Ben-Bassat, Meyer & Tractinsky, 2006; Douneva, Haines & Thielsch, 2015; Katz, 2010; Ling & van Schaik, 2006; Sonderegger et al., 2012). However, goal orientation was only a side product in these previous studies and was not systematically manipulated.

The effect of aesthetics on performance may be stronger in a learning goal than in a performance goal context because the latter one may lead to a ‘tunnel vision’ (Locke & Latham, 2006, p. 266) due to the high stress and pressure (Seijts & Latham, 2005) triggered by performance goal orientation. Individuals who are striving for a performance goal will strongly concentrate on their primary task to come up with a desirable result. Consequently, these individuals may not be affected by secondary cues such as aesthetics. On the contrary, individuals pursuing a learning goal may have stronger intrinsic motivation (Benware & Deci, 1984), and face lower stress and pressure than in a performance goal condition (Seijts & Latham, 2005) because they focus on skill acquisition and not on the assessment of the final result. Thus, they will be prone to marginal influences, such as the aesthetical surroundings.

This would imply a dual-process-model, analogous to theories such as the Elaboration Likelihood Model (ELM; Petty & Cacioppo, 1986) or the Heuristic-Systematic Model of information processing (HSM; Chaiken, Liberman & Eagly, 1989). When pursuing learning goals, information would be heuristically processed on a peripheral route in which superficial surrounding factors (such as aesthetics) play an important role. Performance goals do not leave any space for influencing peripheral cues (e.g., aesthetics) and are thus systematically pursued on the central route–people only focus on facts to solve the particular task or problem. The aesthetical surrounding in a learning goal context might increase motivation and put users at ease (see Lindgaard, 2007; Sonderegger & Sauer, 2010) which in turn should positively affect performance. Since the final result is secondary in a learning context, individuals prolonging their joyful experience will still achieve learning performance goals. This would make the ‘prolongation of joyful experience’ hypothesis (Sonderegger & Sauer, 2010) a negligible or even positive impact on learning performance (Sonderegger et al., 2014).

### Aim of research and hypotheses

This study investigates the effects of an aesthetic website interface on individual performance. The main objective is to directly manipulate aesthetics and enrich the investigation by a further manipulation of goal orientation. As noted earlier, goal orientation seems to alter the relationship between aesthetics and performance. The main theoretical basis of this study is Bonnardel, Piolat & Le Bigot’s (2011) assumption that aesthetics increases motivation because it puts users at ease (Lindgaard, 2007), and thus boosts performance. Until now, this assumption is lacking a systematic investigation, as is Norman’s (2002, 2004) hypothesis that an aesthetic interface evokes positive emotions fostering performance (see Thielsch & Niesenhaus, 2017).

In addition, because websites may be used to fulfill different types of tasks, we investigate an aesthetic interface’s effect on performance in different types of tasks. On the web, users aim to find specific information or solutions to a given problem or they just seek for inspiration and explore a website (e.g., Iten, Troendle & Opwis, 2018; Van Schaik & Ling, 2009). The effects of aesthetics on performance using a website might depend on the nature of the given task. Thus, in the present study, the effect of aesthetics on performance is investigated using not only typical search tasks (e.g., Tuch et al., 2009; Van Schaik & Ling, 2009), but also a creative and a transfer task (Bonnardel, Piolat & Le Bigot, 2011; Plass et al., 2014; Um et al., 2012). As there are slightly more studies reporting positive effects than negative ones or no effects (Thielsch & Niesenhaus, 2017), we assume a positive influence of interface aesthetics on user performance and thus propose hypotheses according to currently discussed theories on the topic as follows:

Regarding individual search tasks, we hypothesize that there will be only an effect of aesthetics on performance if learning goals are instructed (H1). That is because learning goals also allow for processing superficial surrounding cues (heuristic processing on a peripheral route; Chaiken, Liberman & Eagly, 1989; Petty & Cacioppo, 1986) like aesthetics and render aesthetics influential (see above). A high aesthetic interface then positively influences performance due to increased motivation (Sonderegger & Sauer, 2010). Hence, we statistically hypothesize an interaction effect of aesthetics and goal orientation in search tasks.

Furthermore, there are some promising assumptions (Norman, 2002) and first results (Bonnardel, Piolat & Le Bigot, 2011) that aesthetics may have an impact on performance in creative tasks. Thus, we hypothesize that aesthetics positively influences performance in creative tasks, regardless of goal orientation (H2). The theoretical idea is that a creative task demands for ‘out of the box’-thinking (e.g., Weisberg, 2009) and thus promotes heuristic processing on the peripheral route which makes aesthetics influential. Aesthetics will again boost performance due to increased motivation (Sonderegger & Sauer, 2010). Since learning goal orientation is positively related to creativity (Gong, Huang & Farh, 2009; Hirst, van Knippenberg & Zhou, 2009), we assume that a positive effect of aesthetics on performance in creative tasks increases in a learning goal context (H3). The idea is, that learning goals allow for more processing on the peripheral route (see above), which increases the influence of aesthetics. Statistically spoken, we predict a main effect of aesthetics as well as an interaction effect of aesthetics and goal orientation in creative tasks.

Similar to creative tasks, there are some first promising results concerning the effect of aesthetics on transfer performance (Plass et al., 2014 (study 2); Um et al., 2012). Heidig, Müller & Reichelt (2015) presume design aesthetics to positively influence a learner’s intrinsic motivation, which is in turn necessary in a transfer task (see Barnett & Ceci, 2002, for a detailed overview on transfer). Therefore, we hypothesize that aesthetics has a positive effect on performance in transfer tasks (H4) due to increased motivation (see above; Sonderegger & Sauer, 2010). Additionally, learning goals are positively related to metacognition and performance in transfer tasks (Ford et al., 1998). Thus, we also hypothesize that the positive effect of aesthetics on performance in transfer tasks increases in a learning goal context (H5), again because learning goals might facilitate cognitive processing via the peripheral route and thus make aesthetics influential. In sum, we expect a main effect of aesthetics and an interaction effect of aesthetics and goal orientation in transfer tasks.

In order to have it clearly arranged, the hypotheses read as follows:

H1: Participants working with an aesthetic interface show a higher performance with regard to accuracy in a search task than participants working with an unaesthetic interface if they are pursuing a learning goal (in contrast to a performance goal).

H2: Participants working with an aesthetic interface show a higher performance with regard to accuracy in a creative task than participants working with an unaesthetic interface.

H3: Pursuing learning goals increases the positive effect of aesthetics on performance in a creative task.

H4: Participants working with an aesthetic interface show a higher performance with regard to accuracy in a transfer task than participants working with an unaesthetic interface.

H5: Pursuing learning goals increases the positive effect of aesthetics on performance in a transfer task.

## Method

### Sample

The sample consisted of 331 German-speaking participants, including 208 females (62.84%) and 123 (37.16%) males. Their age ranged from 16 to 81 years (*M* = 48.08, *SD* = 14.41). On average, the participants had used the Internet for 16.51 years (*SD* = 4.69). They reported using the Internet on average 3.11 h (*SD* = 3.09) per day, mainly to send or receive e-mails (316; 95.47%), to use search engines (309; 93.35%) and to find specific information or offers (292; 88.22%). Thus, they were familiar with the types of tasks focused in the present study. In the sample, 3.63% (*n* = 12) reported professional work experiences in web design (1.21% main occupation; 2.42% part-time job). Yet such experienced user did not differ in the judgment of aesthetics (*F*(2, 328) = 0.99, *p* = .38, *η*^2^ = .01), and thus were not excluded from further analysis. Regarding medicine, 20.24% (*n* = 67) were experienced (10.27% main occupation; 9.97% part-time job). Furthermore, 249 (75.23%) had achieved a university-entrance diploma (the German ‘(Fach-)Abitur’). Participants were invited via the online panel ‘PsyWeb’ (https://psyweb.uni-muenster.de) and received an invitation link via e-mail. They took part voluntarily, on an anonymous basis, and were incentivized by the possibility to receive a research report and by the chance of winning one of three book vouchers amounting to 50 €, 25 € or 15 €. In total, 12 vouchers (three in each of the four experimental groups; 4 × 50 €, 4 × 25 €, 4 × 15 €) were given.

#### Drop-out rates and excluded data

Initially, 613 potential participants clicked on the invitation link. Among those, 418 finished the study and agreed to have their data used and analyzed. The incomplete data of the 195 participants (31.81%), who did not finish the study, were not included into the analysis. Participants, who did not finish the study (*M*_age_ = 50.24, *SD*_age_ = 15.08; 51.85% female; 75.93% German ‘(Fach-)Abitur’), did not significantly differ from those, who finished the study (*M*_age _ = 47.62, *SD*_age_ = 14.94; 61.72% female; 77.27% German ‘(Fach-)Abitur’), with respect to age, *t*(290.55) = 1.88, *p* = .06, *g* = 0.21, gender, *χ*^2^(2) = 5.23, *p* = .07, and formal education, *χ*^2^(4) = 0.62, *p* = .96. Furthermore, the data of 31 participants (5.06%) could not be included into the analysis due to dyschromatopsia. While 12 participants reported dyschromatopsia in a self-evaluative question, 19 failed the Ishihara picture test (Ishihara, 1972). Moreover, a lack of technical requirements was decisive to have eight persons (1.31%) excluded. Finally, the data of 48 participants (7.83%) had to be excluded because they used additives, e.g., search engines, during the tasks. In total, the drop-out rate accounts to 46.00%.

### Materials and experimental manipulations

The two independent variables, aesthetics and goal orientation, were directly manipulated.

#### Manipulation of aesthetics

**Figure 1:**
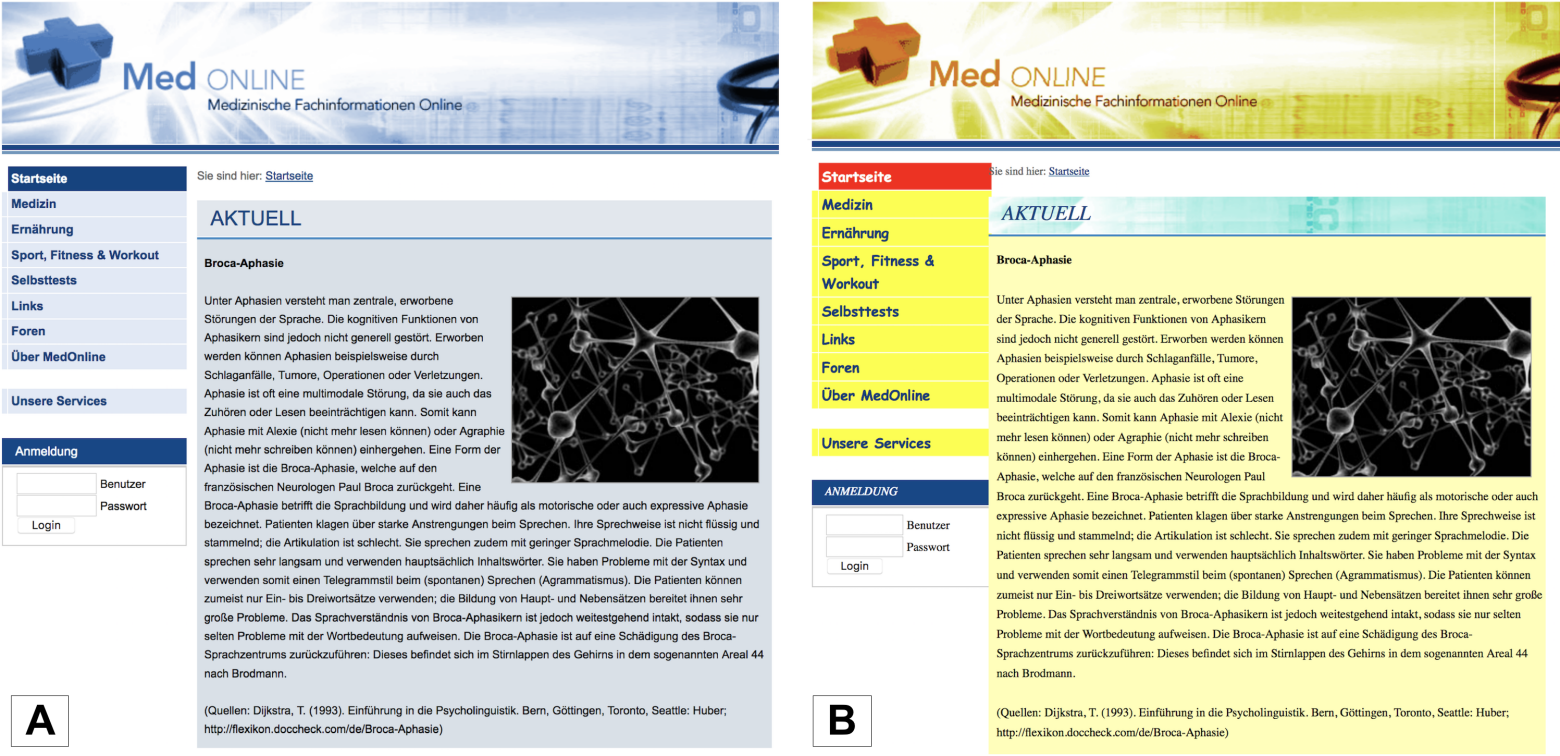
Operationalization of aesthetics - (A) screenshot aesthetic version, (B) screenshot unaesthetic version. Screenshots are trimmed due to copyright reasons; full versions can be requested via the corresponding author.

We aimed at manipulating aesthetics in a maximum possible amount, especially via the facet colorfulness (see Moshagen & Thielsch, 2010), to elicit substantial effects and avoid problems caused by a weak treatment (Douneva, Haines & Thielsch, 2015). Reinecke & Gajos (2014) showed that Germans prefer less colorful websites which is why the unaesthetic interface should be very varicolored. On the contrary, the aesthetic interface should only consist of two or three different colors belonging to one color scheme. Moshagen & Thielsch (2010) showed that the interplay of blue, white and black is seen as an appealing color scheme. Furthermore, a mixture of different fonts should be used in the unaesthetic condition to interfere with consistency (De Angeli, Sutcliffe & Hartmann, 2006) and craftsmanship (Moshagen & Thielsch, 2010). Two pretested interfaces, which were formerly created for aesthetics research at the Department of Psychology, University of Münster, were used. Both websites dealt with medical information, were named “Med Online” and were of the same usability and content. They differed in color (Cyr, Head & Larios, 2010; Ling & van Schaik, 2002; Moshagen & Thielsch, 2010), font consistency (see above), and composition of objects in header and main area of the website (Schenkman & Jönsson, 2000;  Zheng et al., 2009) to meet the above-mentioned criteria. Figure 1 shows screenshots of the two stimuli.

#### Manipulation of goal orientation

Goal orientation was manipulated by different task instructions. Learning goal orientation was induced via keywords such as “chance”, “possibility to learn”, “try out”, “useful to learn” (Elliott & Dweck, 1988; Utman, 1997), which were marked in bold face. The participants were informed that they only needed to finish the study to participate in the lottery of vouchers. This should trigger intrinsic motivation. By contrast, performance goal orientation should be emphasized by keywords like “test”, “be better than others” and “consequences” (Elliott & Dweck, 1988; Utman, 1997), which were likewise highlighted in bold face. Furthermore, winning a voucher was connected to the performance, so that the participants were told that only the first, second and third place would win a voucher of 50 €, 25 €, 15 €, respectively. Thus, these participants should be extrinsically motivated. The different instructions had nearly the same length (Task 1: 84 words (learning goal) vs. 82 words (performance goal); Task 2: 43 vs. 41; Task 3: 95 vs. 94) and contained the same important information in both conditions. For a closer look on the instructions, readers are referred to the online supplement, in which complete instructions are provided, both in the original German and translated into English*.*

### Measures

#### Manipulation checks

For the aesthetics treatments, two different manipulation checks were used. First, the participants were asked to rate the design of the given website with the help of the VisAWI (Visual Aesthetics of Websites Inventory; Moshagen & Thielsch, 2010) which consists of 18 items on four scales: simplicity (*α* = .89), diversity (*α* = .86), colorfulness (*α* = .88), and craftsmanship (*α* = .87). Judgments had to be given on a 7-point Likert scale ranging from 1 (strongly disagree) to 7 (strongly agree). Overall Cronbach’s alpha amounted to *α* = .95. In addition, the two website designs of the study were directly contrasted, and the participants chose the design they found more attractive in a paired comparison.

Goal orientation was manipulated via instructions (see above). The goal orientation manipulation checks consisted of two survey items. In the first, the two possible instructions of the first task were contrasted, and participants were asked to choose the instruction that more strongly emphasized the aim of learning development (learning goal). In the second, the two possible instructions of the second task were presented in a similar paired comparison. This time, participants were asked to choose the instruction that more strongly focused on an achieved accomplishment (performance goal).

Our manipulations were aimed directly at goal orientation and particularly website aesthetics. Website aesthetics shows significant overlap with perceptions of website content and usability (for an overview see Thielsch, Blotenberg & Jaron, 2014). Thus, as further manipulation check variables, content and usability were also rated on a 7-point Likert scale ranging from 1 (strongly disagree) to 7 (strongly agree). The content items were extracted from the Web-CLIC questionnaire (Thielsch & Hirschfeld, in press), which originally consists of 12 items (.92 ≤ *α* ≤ .94) on four scales: clarity (*α* = .83), likeability (.92 ≤ *α* ≤ .93), informativeness (.89 ≤*α* ≤ .91), and credibility (.93 ≤ *α* ≤ .95). To avoid possible demotivating effects caused by too many items, the Web-CLIC short scale consisting of four items was used: “I enjoy reading the website”, “The contents of the website are clearly presented”, “The website is informative”, and “I can trust the information on the website.” Cronbach’s alpha of the short scale amounted to *α* = .80. Usability was measured with the best fitting item of the SUS (“I thought the system was easy to use”; Brooke, 1996; German translation by Rauer, 2011).

#### Dependent variables: performance tasks

Three different tasks were used to measure performance. The tasks were pretested with 12 participants invited via the same online panel ‘PsyWeb’ (https://psyweb.uni-muenster.de/). Their age ranged from 24 to 63 years (*M* = 48.25, *SD* = 13.16); ten (83.33%) were female and two (16.67%) were male. Participants took part voluntarily, anonymously, received a research report and had the possibility to win one of three book vouchers (in the amount of 50 €, 25 €or 15 €) at the end of the whole study. As a result of the pretest, the first performance task was made more difficult, in order to prevent ceiling effects. From 8 possible points, participants achieved on average 7.17 (*SD* = 1.27) in the pretest. Furthermore, the scenario of the creative task was redrafted, since some participants noted that it was unclear.

In the first task, participants were given a text about Broca aphasia (adapted from Dijkstra, 1993) and https://de.wikipedia.org/wiki/Sprechst%C3%B6rung; http://flexikon.doccheck.com/de/Broca-Aphasie), consisting of 296 words. The text dealt with information on symptoms, development and anatomic features of Broca aphasia as well as the demarcation to other similar dysfunctions. Participants were requested to answer five open questions after having read the text. The answers to the questions could be found in the text itself. One point was given for every correct answer, except for one unique question, in which four points could be achieved. This led to a minimum of 0 and a maximum of 8 points. In the main study, participants achieved on average 6.07 points (which is equivalent to a difficulty of .76).

The second task was of creative nature. The participants were confronted with the scenario that a person was in hospital with an unclear diagnosis and was nervously waiting for an important examination the day after tomorrow. The participants were instructed to generate activities with which they could distract the person during the day before the examination. In the creative task, one point was given for every reasonable answer. These points were counted, building a sum score. On average, 4.23 ideas were generated in the main study.

The third task was a transfer task. The participants were provided with a text dealing with Wernicke aphasia, consisting of 153 words (adapted from Dijkstra, 1993 and http://flexikon.doccheck.com/de/Wernicke-Aphasie). The text was relatively analogous to the text about Broca aphasia they read earlier with regard to structure and content. They were given a written speech example (adapted from Dijkstra, 1993) of a patient suffering from aphasia and should diagnose the right type of aphasia (Broca versus Wernicke aphasia). Participants were awarded with one point for the correct answer, the maximum point score in this task (Minimum = 0 points). In the main study, 44 percent of all participants solved this task (task difficulty of .44).

For each of the three tasks, detailed information on the scoring can be found in the coding scheme within the data package (provided as online supplement). Open answers were coded by one of the authors. Besides accuracy, response times were measured for all three performance tasks via timestamps.

#### Control variables and further variables

Since the tasks address more strongly to fluid than to crystallized intelligence, which decreases with age (Horn & Cattell, 1967), age was included as a control variable. Medical interest was measured by the single item “I find medical issues interesting.”, which was rated on the 7-point Likert scale described above. Moreover, medical experience was measured with a single item in which the participants stated whether they had professional working experience in the medical industry. They could choose between “Yes, as a full-time job”, “Yes, as a part-time job” or “No” as answer possibilities. Formal education served as proxy for cognitive skills, which are also possible influencers of performance (e.g., Kramer, 2009). The participants indicated their highest degree of formal education out of the answer possibilities “(Fach-)Abitur” (equivalent to university-entrance diploma), “Realschulabschluss”, “Hauptschulabschluss”, “No graduation” and “Other” in the demographic check.

Motivation was measured with a pre-/post-task single-item measurement. The pre-task motivation-item was “I am motivated to work on this task”, while the post-task-motivation-item was “I was motivated working on this task”. Participants had to rate these statements on a 7-point Likert scale ranging from 1 (strongly disagree) to 7 (strongly agree). This way of global motivation assessment was adapted from cognitive research (e.g., Brose et al., 2012). Mood was measured with the Smiley measurement of Jäger (2004). Participants had to rate their current mood on a 5-point smiley scale ranging from a very sad to a very happy smiley. In a series of two studies, Jäger (2004) provided evidence for unidimensionality and equidistance of this scale as well as high correlations with the German version of the PANAS scale (.75 ≤ *r* ≤ .89). Stress was measured with a single-item measurement adapted from typical global stress measures (e.g., Childs, Bershad & De Wit, 2016; Howell et al., 2014). Participants were asked to rate the stress caused by each task with the statement “I perceived this task to be stressful” on a 7-point Likert scale again ranging from 1 (strongly disagree) to 7 (strongly agree).

### Study design and procedure

The web-based experiment featured a 2 (aesthetics: high vs. low) ×2 (goal orientation: learning vs. performance goals)-between-subject design. Due to the exclusions, the four groups did not consist of the exact same number of participants (aesthetic interface/learning goals: *n* = 90; aesthetic interface/performance goals: *n* = 67; unaesthetic interface/learning goals: *n* = 79; unaesthetic interface/performance goals: *n* = 95). Yet the four groups did not differ regarding the central demographic variables age, *F*(3, 327) = 0.82, *p* = .48, *η*^2^ = .01, gender, *χ*^2^(3) = 3.71, *p* = .30, and formal education, *χ*^2^(3) = 1.45, *p* = .69.

**Figure 2:**
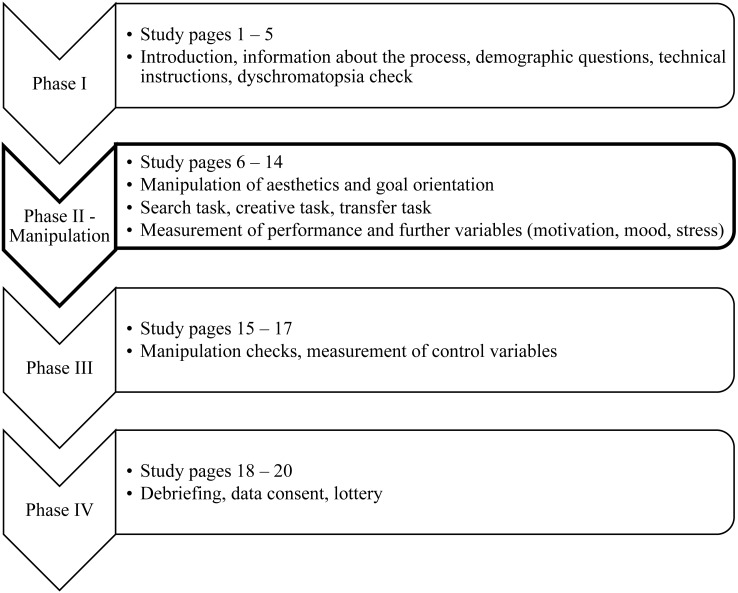
Simplified schedule of the study.

The experiment was structured in four phases (see Fig. 2). On average, the participants needed 1,693.80 (*SD* = 1640.58) seconds, which is about 28 min, to complete the study.

#### Phase I

Participants received an e-mail via the German online panel ‘PsyWeb’ with a short invitation text and the link to the study. It was labeled as a study measuring the processing of medical information. Having clicked on the link, the participants were randomly assigned to one of the four groups. After an introduction web page containing informed consent information on the purpose and focus of the study, anonymity, compensation, and age restriction (participants younger than 14 years were not allowed), those that agreed to participate were provided with more detailed information on the process of the present study on a second page. The third page dealt with demographic questions. Besides standard demographic questions, Internet use, work experience in web design as well as medical experience were asked. Afterwards, participants received technical instructions; for instance, they were required not to use mobile phones and small tablets, since a minimum screen size and a keyboard were necessary. Dyschromatopsia was checked on the fifth page via three plates of the Ishihara picture test (Ishihara, 1972) and a self-evaluative item.

#### Phase II

Subsequently, participants were asked to rate their current mood and pre-task motivation after they were given the instructions for the first task (having either a learning or performance goal), and before the actual task started on the following page. In both conditions, participants were asked to read the text and to answer the five questions. The text on Broca aphasia appeared in the center of the either aesthetic or unaesthetic website. The website itself was embedded into the study via an iframe. The five questions were displayed below the website. Mood, post-task motivation and stress were rated on the next page, and participants indicated whether they used supplements like search engines in a closed question. Next, the preparation for the creative task followed. Again, participants rated their motivation before the second task on a page with a neutral instruction. The manipulative instruction, including either a learning or performance goal, only appeared on the following page, which contained the second treatment. On this page, a neutral scenario appeared on the web page, describing a person in hospital in need of distraction (see ‘Materials’). Underneath, the participants were invited to generate their ideas in an open text field. The exact instruction varied, depending on the goal orientation condition. Similar to the first task, the page with items regarding mood, stress, post-task motivation and supplements followed. The third and final task was introduced on the next page. Again, participants were given their particular instruction depending on the goal orientation condition and were asked to rate their pre-task motivation. The construction of the actual task on the next page was very analogous to the first task. A text about Wernicke aphasia appeared in the center of the website and the written speech example for the diagnosis was shown below. At the very bottom of the page, the participants typed in their diagnosis in an open text field. Unlike the first task, the website was embedded as a screenshot and not as an iframe. The reason was that the participants were not allowed to surf on the website because then they would have been able to see the text about Broca aphasia again, which is incongruous with the idea of a transfer task.

#### Phase III

After the typical post-treatment page with items on mood, stress, post-task motivation and additives, a section of different questionnaires and scales followed. First, the participants rated the design of the website with the help of the VisAWI (Moshagen & Thielsch, 2010). The 18 items occurred in a randomized order. In order to prevent possible halo effects, the design the participants had to deal with was shown again on top of the page. On the next page, the participants answered items regarding the control variables of website content, usability, medical interest, and two additional measures (not pertinent to the present study). The manipulation checks were conducted on the next page via paired comparisons.

#### Phase IV

Finally, the participants were debriefed regarding the real aim of the study, namely the investigation of interface aesthetics and performance. They were informed about the different groups and were able to comment on the study in an open text field. To avoid unpleasant feelings, it was clarified that the performance goal condition could sound stressful and harsh for some people, but that this was part of the manipulation. On the following page, the participants stated whether they gave consent on the analyses of their data. The last page dealt with the compensation. Thus, participants could leave their e-mail address if they were interested in winning one of the book vouchers. Furthermore, they could download a short research report as incentive. The research report, consisting of 1.5 pages, gave a short insight into the theoretical background and the aim of the study.

## Results

Results were calculated with the statistics program R (Version i386 3.2.0) and its additional packages “Rcmdr”, “car”, “psych” and “fmsb”. The level of significance was *α* = .05. The adjusted *R* ^2^ served as effect size for the regressions.

### Data preparation

In our analyses, we performed regression analysis with dummy-coded treatment levels. Compared to a classical ANOVA, this way of analysis has advantages when it comes to the interpretation of data, as regression analyses allow for a direct interpretation of effects while an ANOVA would require further post-hoc tests^1^. 1We also calculated the according MANCOVA and found the same main results for the aesthetic treatment.Thus, in the final data set, the aesthetic interface was encoded “1”, while the unaesthetic one was encoded “0”. Similarly, learning goal orientation was encoded “1” and performance goal orientation “0”. When measuring performance, false or missing answers were encoded “0” and correct ones “1”. For each task and each participant, a sum score was calculated. The evaluation was undertaken task for task and not person for person, in order to avoid halo effects (e.g., Asch, 1946). The sum scores ranged from 0–8 for the first task, 0–21 for the second task, and 0–1 points for the third task. Means were calculated for the short version of the Web-CLIC and the VisAWI. Response times were calculated for each task by subtracting the starting timestamp from the ending timestamp. These values were converted into seconds and were then logarithmized. Both medical experience and formal education were dummy coded.

### Manipulation checks

By all measures, participants considered the aesthetic interface to be significantly more attractive than the unaesthetic interface. First, in a linear regression (*R*^2^ = .29), the aesthetic interface (*M* = 4.46, *SD* = 0.93) had a significantly higher VisAWI score than the unaesthetic interface (*M* = 3.13, *SD* = 1.14), *β* = 1.33, *t*(329) = 11.59, *p* < .001, *g* = 1.27.^2^ 2This was found for each single scale of the VisAWI as well, with particularly large differences on simplicity, colorfulness, and craftmanship.Moreover, 260 (78.55%) participants confirmed the aesthetic interface to be more attractive than the unaesthetic one in a paired comparison, which significantly differs from coincidence (50%), *χ*^2^(1) = 107.92, *p* < .001. Finally, the mean score of the aesthetic version matches the cut point for “good” according to the recommendations for the VisAWI (given by Hirschfeld & Thielsch, 2015).

Regarding goal orientation, 282 (85.20%) participants confirmed that the learning goal instruction emphasized individual learning development and 216 (65.26%) participants confirmed that the performance goal instruction stressed output/performance in a paired comparison. Both ratings were significantly beyond coincidence (50%), learning: *χ*^2^(1) = 164.02, *p* < .001 and performance: *χ*^2^(1) = 30.82, *p* < .001.

Although not manipulated, content und usability ratings differed significantly depending on the aesthetic treatment, *t*(329) = 6.10, *p* < .001, *g* = 0.67 and *t*(319.17) = 4.87, *p* < .001, *g* = 0.53. Thus, the participants facing the aesthetic treatment rated both content (*M* = 4.76, *SD* = 0.94) and usability (*M* = 5.25, *SD* = 1.05) higher than those who had to deal with the unaesthetic interface (content: *M* = 4.10, *SD* = 1.04; usability: *M* = 4.59, *SD* = 1.39). This close connection in users’ perception of content, usability and aesthetics is a consistent finding in HCI literature (e.g., De Angeli, Sutcliffe & Hartmann, 2006; Lee & Koubek, 2010; Lee & Koubek, 2012; Moshagen, Musch & Göritz, 2009; Thielsch, Blotenberg & Jaron, 2014) and did not yield to any adjustments for further analyses.

### Performance tasks

The dependent variables, namely the accuracy and response times of the three performance tasks, were treated separately in this analysis. Non-parametric correlations (Kendall’s Tau) were used to analyse their overlap: Table 1 shows the correlations for the three performance tasks regarding accuracy and logarithmized response times. According to Cohen (1992), the correlations were small to medium. All correlations were significant.

**Table 1:** Correlation matrix of the three performance tasks.

	Search task	Creative task	Transfer task
Search task	–	*r*(329) = .13^*^	*r*(329) = .21^***^
Creative task	*r*(329) = .32^***^	–	*r*(329) = .10^*^
Transfer task	*r*(329) = .39^***^	*r*(329) = .29^***^	–

**Notes.**

Correlations regarding accuracy were displayed above the diagonal; correlations regarding logarithmized response times were displayed below the diagonal.

Significance codes:

*** *p* < .001.

* *p* < .05.

#### Search task

Considering performance (accuracy) on the first (search) task, we assumed an interaction effect of aesthetics and goal orientation (H1). Table 2 shows the descriptive statistics for this. On a descriptive level, the average number of correct answers was a little higher in both the aesthetic and the performance goal conditions.

**Table 2:** Means (and standard deviations) of correct answers in the search task, separated for conditions.

	Goal orientation		
Aesthetic treatment	Learning	Performance	Overall
Aesthetic	6.06 (1.65)	6.40 (1.56)	6.20 (1.62)
Unaesthetic	5.67 (1.63)	6.19 (1.53)	5.95 (1.59)
Overall	5.88 (1.65)	6.28 (1.54)	6.07 (1.61)

**Notes.**

*N* = 331; scale: 0–8.

Due to having count data, a Poisson regression (*Nagelkerke R* ^2^ = .18) was calculated for the first task. In this regression, we controlled for age, medical interest, medical experience, formal education, motivation, stress and mood. Neither the main effects of aesthetics (*β* = 0.02, *e*^*β*^ = 1.02, *SE* = 0.06, *p* = .78) and goal orientation (*β* =  − 0.10, *e*^*β*^ = 0.90, *SE* = 0.06, *p* = .11), nor the interaction effect (see H1; *β* = 0.06, *e*^*β*^ = 1.06, *SE* = 0.09, *p* = .52) turned out to be significant. This last result did not support the first hypothesis (H1).

Table 3 shows the descriptive statistics of the response times in the search task. For this table, the means were trimmed by 5% to eliminate extreme values in response time which cannot be controlled for, especially in an online study. It shows that participants were descriptively the fastest in the learning goal condition and when dealing with an unaesthetic interface.

**Table 3:** 5%-trimmed means (and standard deviations) of response times (in seconds), in the search task, separated for conditions.

	Goal orientation		
Aesthetic treatment	Learning	Performance	Overall
Aesthetic	355.15 (198.71)	365.90 (257.89)	359.16 (225.46)
Unaesthetic	345.38 (365.56)	369.18 (264.06)	356.07 (313.28)
Overall	349.87 (288.26)	365.43 (260.72)	356.82 (274.83)

**Notes.**

*N* = 331.

A multiple regression (*R*^2^ = .06) with logarithmized response times and age, medical interest, medical experience, formal education, motivation, stress and mood as control variables was calculated with response time as the dependent variable. Again, neither the main effects of aesthetics (*β* = 0.02, *SE* = 0.08, *p* = .83) and goal orientation (*β* =  − 0.07, *SE* = 0.08, *p* = .38), nor the interaction effect (*β* = 0.02, *SE* = 0.11, *p* = .88) were significant.

#### Creative task

Regarding the second task, we assumed a main effect of aesthetics (H2) and an interaction effect of aesthetics and goal orientation (H3) on performance. Table 4 shows the descriptive statistics for performance in the creative task. Descriptively, the participants facing an unaesthetic interface and having learning goals came up with the highest number of ideas.

**Table 4:** Means (and standard deviations) of the number of ideas in the creative task, separated for conditions.

	Goal orientation		
Aesthetic treatment	Learning	Performance	Overall
Aesthetic	4.01 (2.00)	3.99 (3.01)	4.00 (2.47)
Unaesthetic	4.52 (2.99)	4.37 (3.34)	4.44 (3.18)
Overall	4.25 (2.52)	4.21 (3.20)	4.23 (2.87)

**Notes.**

*N* = 331, scale: 0–21.

For this task, a Poisson regression (*Nagelkerke R*^2^ = .13) with the control variables age, medical interest, medical experience, formal education, motivation, stress and mood was calculated. Again, neither the main effects of aesthetics (see H2; *β* =  − 0.09, *e*^*β*^ = 0.91, *SE* = 0.08, *p* = .26) and goal orientation (*β* = 0.03, *e*^*β*^ = 1.03, *SE* = 0.07, *p* = .66), nor the interaction effect (see H3; *β* =  − 0.03, *e*^*β*^ = 0.97, *SE* = 0.11, *p* = .82) were significant. These results do not support the second and the third hypothesis.

Taking into account response time as the dependent variable, Table 5 shows the descriptive statistics for the creative task. As above, the means were trimmed by 5%. The group facing the aesthetic and learning goal orientation treatment was descriptively the fastest.

**Table 5:** 5%-trimmed means (and standard deviations) of response times (in seconds), in the creative task, separated for conditions.

	Goal orientation		
Aesthetic treatment	Learning	Performance	Overall
Aesthetic	204.29 (214.92)	343.97 (323.80)	261.10 (275.29)
Unaesthetic	247.12 (225.16)	257.81 (410.77)	250.28 (339.26)
Overall	220.99 (219.84)	290.00 (377.33)	254.19 (310.11)

**Notes.**

*N* = 331.

Again, a multiple regression (*R*^2^ = .11) with logarithmized response times and age, medical interest, medical experience, formal education, motivation, stress and mood as control variables was calculated. Both the main effect of aesthetics (*β* = 0.21, *SE* = 0.12, *p* = .08) and the interaction effect (*β* =  − 0.30, *SE* = 0.17, *p* = .07) were not significant. The main effect of goal orientation (*β* =  − 0.09, *SE* = 0.12, *p* = .45) was not significant either.

#### Transfer task

For the third task, we hypothesized a main effect of aesthetics (H4) and an interaction effect of aesthetics and goal orientation on performance (H5). Table 6 reports relative frequencies of correct answers in the transfer task. Overall, the aesthetic and learning goal orientation treatment yielded a descriptively higher percentage of correct answers, and considering the single experimental groups, the group facing the aesthetic treatment and pursuing performance goals had descriptively the highest proportion of correct answers.

**Table 6:** Relative frequencies of correct answers in the transfer task (percentages), separated for conditions.

	Goal orientation		
Aesthetic treatment	Learning	Performance	Overall
Aesthetic	44	48	46
Unaesthetic	46	41	43
Overall	45	44	44

**Notes.**

*N* = 331.

Due to having binomial data, a logistic regression (*Nagelkerke R*^2^ = .19) was calculated to test hypotheses four and five. In this regression, we controlled for age, medical interest, medical experience, formal education, motivation, stress and mood. The main effects of aesthetics (see H4; *β* = 0.13, *OR* = 1.14, 95% CI (OR) [0.57–2.29], *SE* = 0.35, *p* = .71) and goal orientation (*β* = 0.16, *OR* = 1.18, 95% CI (OR) [0.60–2.32], *SE* = 0.34, *p* = .63, as well as the interaction effect (see H5; *β* =  − 0.25, *OR* = 0.78, 95% CI (OR) [0.29–2.05], *SE* = 0.49, *p* = .61) were not significant. These results did not support the fourth and fifth hypothesis.

Table 7 depicts the descriptive statistics for response times in the transfer task. Means were again trimmed by 5%. Descriptively, an unaesthetic interface and a performance goal orientation resulted in a faster response time.

**Table 7:** 5%-trimmed means (and standard deviations) of response times (in seconds), in the transfer task, separated for conditions.

	Goal orientation		
Aesthetic treatment	Learning	Performance	Overall
Aesthetic	134.90 (91.53)	143.51 (90.74)	138.57 (90.94)
Unaesthetic	142.66 (135.90)	127.33 (111.45)	132.46 (123.07)
Overall	136.65 (114.24)	132.82 (103.21)	134.73 (108.86)

**Notes.**

*N* = 331.

A multiple regression (*R*^2^ = 0.03) with logarithmized response times and age, medical interest, medical experience, formal education, motivation, stress and mood as control variables was calculated. No significant main effects of aesthetics (*β* = 0.13, *SE* = 0.09, *p* = .16) and goal orientation (*β* = 0.04, *SE* = 0.09, *p* = .68) could be unveiled. The interaction effect (*β* =  − 0.10, *SE* = 0.13, *p* = .44) did not show any significance either.

As we found none of the expected main effects neither of aesthetics nor of goal orientation on performance, further analyses on potential moderators and mediators could not be performed.

## Discussion

The research question of this study was whether an aesthetic interface could enhance performance in three types of tasks (search task, creative task, transfer task) under consideration of goal orientation (learning versus performance goal) as a possible moderator. Unlike several studies showing either a positive (e.g., Douneva, Jaron & Thielsch, 2016; Miller, 2011; Pomales-Garcia, Liu & Mendez, 2005; Sonderegger & Sauer, 2010; Strebe, 2016; Tuch et al., 2009; Um et al., 2012; Van Schaik & Ling, 2008) or a negative effect (Sauer & Sonderegger, 2011; Sonderegger et al., 2014; Van Schaik & Ling, 2009), the regressions in this study did not reveal any significant main effects on performance measured by both accuracy and response times by aesthetics or goal orientation, and no significant interaction effects of the two. Thus, hypotheses H1–H5 were not confirmed. These results add theoretical weight to a growing line of studies in other contexts in which no effect of aesthetics on performance was detected (e.g., Ben-Bassat, Meyer & Tractinsky, 2006; Chawda et al., 2005; Douneva, Haines & Thielsch, 2015; Hall & Hanna, 2004; Ilmberger, Schrepp & Held, 2008; Katz, 2010; Ling & van Schaik, 2006; Nakarada-Kordic & Lobb, 2005; Sonderegger et al., 2012; Thüring & Mahlke, 2007; Tractinsky, Katz & Ikar, 2000). According to the present study, one could conclude that there is no notable effect of design aesthetics (in terms of a combined editing of color, font and object composition) on performance in virtual tasks. Unlike our expectations, goal orientation did not moderate the effect of aesthetic design aspects on performance. Thus, our experiment found no evidence that learning goals might increase (or even trigger) a positive effect of website aesthetics on performance for either search, creative, or transfer tasks. Yet unlike in some few other studies, we showed no *negative* effect of aesthetics on performance. Thus, from an organizational perspective, it is likely of no drawback to create an aesthetic interface when a new interface is needed anyway, which would have a positive influence on subjective outcomes as several studies showed (e.g., Bargas-Avila & Hornbæk, 2011; Hassenzahl & Tractinsky, 2006; Thielsch, Blotenberg & Jaron, 2014).

In sum, our study could not find evidence for the ‘increased motivation’ hypothesis with respect to increased performance in the given tasks caused by aesthetic design (Sonderegger & Sauer, 2010). Furthermore, there were no indications for the ‘positive affect mediation’ hypothesis (Norman, 2002; Norman, 2004) and the ‘prolongation of joyful experience’ hypothesis (Sonderegger & Sauer, 2010). All in all, the regressions as well as descriptive results contribute to the impression that there is neither a notable positive nor negative effect of website aesthetics on performance in search, creative, and transfer tasks. Furthermore, any evidence for learning goal orientation being a triggering or reinforcing moderator is lacking.

### Limitations and future research

Some limitations must be considered when interpreting the results of the present study, most of them sketching avenues for future research. First, even as our manipulation checks were successful, we were not particularly able to determine to what extent participants actually felt more learning- or performance-orientated during the treatment. While the goal orientation manipulation instruction was implemented successfully in the intended manner, future studies should aim at measuring the success of such instructions directly, if possible, with the caveat that the question(s) not interfere with the study design by revealing research hypotheses to the participants.

Second, according to recent research, the impact of aesthetics influences first impressions in particular (e.g., Bölte et al., 2017; Thielsch, Engel & Hirschfeld, 2015; Tuch et al., 2012), and some studies raise doubt about stable, long-lasting effects of aesthetics on usage behavior (Iten, Troendle & Opwis, 2018; Sonderegger et al., 2012). Nevertheless, possible effects of aesthetics on performance might occur in work settings in a long-term design via subjective perceptions, because an unaesthetic design could yield losses in motivation and therefore to decrements of performance. There are some hints that an aesthetic interface might have an influence on motivation in some tasks and under certain circumstances (e.g., Reppa & McDougall, 2015; Sonderegger & Sauer, 2010). In our study, the participants were only dealing with one website for about 10 to 20 min. Therefore, they might have been able to cope for an unaesthetic interface because the end of this task was conceivable. Giving a real-life example, an office worker, who has to deal with an unaesthetic interface for eight hours every weekday, may be more strongly influenced by it than the participants of this study. This is why investigating endurance tasks, using different applications instead of a website, and testing field samples in the future would be a fruitful extension to this research area.

Furthermore, there is one study indicating that aesthetics might only enhance performance in the case of sub-optimal usability (Moshagen, Musch & Göritz, 2009). Yet a recent study of  Minge & Thüring (2018) did not find such an interaction effect. Instead,  Minge & Thüring (2018) suggest that there is an interaction between aesthetics and usability in form of halo-effects of aesthetics on usability before use and vice versa after use. Future research should investigate effects of different levels of aesthetics while varying usability (low vs. high) at the same time to further investigate this matter.

In addition, a shift of perspectives might be useful. Instead of expecting high aesthetics to positively influence variables such as motivation or cognitive ability, one could start studying whether low aesthetics leads to a decline in, for example, motivation. As there are many conflicting studies on aesthetics and performance, it is possible that every participant has a performance capacity, which is an interplay of many different factors, such as cognitive ability, age or interest, but not further improved by high aesthetics. Thus, aesthetics, as a relatively soft factor (compared to cognitive skills for instance), might not have the power to considerably increase the individual performance capacity of web users. However, this performance capacity might be decreased by an unaesthetic interface due to a loss of motivation or irritation. Concerning goal orientation as a possible moderator, one further idea would be to not only look at learning versus performance goals, but also at approach versus avoidance behavior and thus use a more specific perspective (Elliot, 1997; Elliot & Harackiewicz, 1996; Harackiewicz et al., 2000). Effects of aesthetics on approach and avoidance behavior were already investigated by Strebe (2016), showing some first promising results on web user behavior in terms of website retrieval and dwell time.

In general, it might be advisable for future research to focus on directly manipulating, instead of just measuring, variables that are important for one of the three existing hypotheses, be it the ‘increased motivation’ hypothesis (Sonderegger & Sauer, 2010) the ‘positive affect mediation’ hypothesis (Norman, 2002; Norman, 2004) or the ‘prolongation of joyful experience’ hypothesis (Sonderegger & Sauer, 2010). For example, instead of just measuring the current mood (as done in the present study), one could think about mood induction to gain an experimental design. This would then need a laboratory setting due to ethical reasons.

## Conclusion

Contrary to our expectations, an aesthetic web interface did not lead to a higher performance on search, creative, or transfer tasks. Moreover, goal orientation does not seem to be a moderator in this area of research. This study forms a building consensus with many other studies in related HCI contexts showing no effect of aesthetics on performance. Since there are many equivocal studies, new theoretical development and research is of great interest and needed to better understand the effects of aesthetics. At least, as an aesthetic design did not *interfere* with work performance goals, aesthetic interfaces might be implemented due to their positive effects on subjective perceptions of users.

##  Supplemental Information

10.7717/peerj.6516/supp-1 Supplemental Information 1 Data package Includes original instructions and items, their translation into English, raw data (without demographics), and a coding scheme for the open answers.

## Additional Information and Declarations

### Data Availability

The following information was supplied regarding data availability: Raw data is available in a Supplemental File.
