# Supplementary material for: Experimental investigation on the effects of website aesthetics on user performance in different virtual tasks
Source: PeerJ. 2019 Feb 22;7:e6516. doi: 10.7717/peerj.6516 (PMC6388663; doi:10.7717/peerj.6516)
Supplement: Supplemental Information 1 — Includes original instructions and items, their translation into English, raw data (without demographics), and a coding scheme for the open answers. [file peerj-07-6516-s001.zip › Data_Package/Coding_scheme_open_answers_performance_tasks.docx]

**Kodierschema der offenen Fragen / Coding scheme of open questions**

Korrekte Antwort / correct answer = 1

Falsche Antwort / wrong answer = 0

**Aufgabe 1 (Suchaufgabe) / Task 1 (Search-and-find task)**

|  | Als „Korrekt“ gewertet / evaluated as „correct“ | Als „Falsch“ gewertet / evaluated as „wrong“ |
| --- | --- | --- |
| Frage / question 1 | „44“, „Areal 44“ o. Ä. / and others | Alle Antworten , in denen die Zahl „44“ nicht auftauchte / answers in which the number „44“ was not mentioned; „44“ und/oder eine falsche Antwort / „44“ and/or a wrong answer |
| Frage / question 2 | „Alexie“ | „Alexi“, „Aplexie“, „Alexie oder Agraphie“, „Legasthenie“ |
| Frage / question 3 | „Tumor“, „Operationen“, „Verletzungen“, „Schlaganfälle“ (jeweils 1 Punkt), auch in Ordnung: „Apoplex“, „Hirnschlag“ => Synonyme / synonyms | „Unfälle“, „angeboren“, „Betäubungsmittel => andere möglicherweise richtige Dinge, die aber nicht in dem Text standen, da Textverständnis und nicht Vorwissen getestet wurde / further possibly correct triggers of Broca aphasia, which were not treated in the text, to prevent a huge influence of previous knowledge |
| Frage / question 4 | „Sprachverständnis“/“Wortbedeutung“, auch in Ordnung: „Sprachverstehen“, „verstehen, was gesagt wird“ | „Sprachvermögen“, „kognitive Funktionen“, „zuhören“ |
| Frage / question 5 | „Telegrammstil“, „Probleme mit der Syntax“ | „Sprechen“, „Sprachstörung“, „langsames Sprechen“ |

**Aufgabe 2 (kreative Aufgabe) / Task 2 (Creative task)**

„Eis/Kuchen/… essen“ = 1 Punkt (nicht für jedes Lebensmittel 1 Punkt; not 1 point for every kind of food)

„Gespräch über Krankheit, mit Arzt ...“ = 0 Punkte (keine Ablenkung / no necessary distraction)

„Sport (wenn nicht näher beschrieben; ausgenommen Spaziergänge o. Ä. / if not further described, except for going for a walk etc.)“ = 0 Punkte, da größtenteils mit Armbruch nicht möglich / 0 points because not doable with a fracture of the arm

„Wein/Alkohol“ / „wine / alcohol“ = 0 Punkte, da vor Untersuchung und im Krankenhaus fragwürdig / 0 points because that is disputable in a hospital)

**Aufgabe 3 (Transferaufgabe) / Task 3 (Transfer task)**

| Als „Korrekt“ gewertet / evaluated as „correct“ | Als „Falsch“ gewertet / evaluated as „wrong“ |
| --- | --- |
| „Broca“, „Broca-Aphasie …“ | „nicht Wernicke“, „Brocca“, „Wernicke“, „die erste“ |
