# Supplementary material for: Experimental investigation on the effects of website aesthetics on user performance in different virtual tasks
Source: PeerJ. 2019 Feb 22;7:e6516. doi: 10.7717/peerj.6516 (PMC6388663; doi:10.7717/peerj.6516)
Supplement: Supplemental Information 1 — Includes original instructions and items, their translation into English, raw data (without demographics), and a coding scheme for the open answers. [file peerj-07-6516-s001.zip › Data_Package/Instructions_English_Translation.docx]

**Instructions**

**Page 1**

| Welcome & introduction  **Study on reading and processing of medical information**  **Dear participant,**  thank you very much for your decision to participate in this study. We are conducting this study as part of a research project in Psychology at Westfälische Wilhelms-University Münster. In this study you will be asked to differentiate between medical subjects. However, you will not need any previous knowledge to answer the questions. All data obtained in this study are of course not analyzed on an individual level but summarized for research purposes, and, furthermore, are recorded anonymously.  The study lasts about 25-30 minutes.  At the end of the study you have the chance to win one of three gift cards for books worth €50, €25 or €15. If you wish to participate in the lottery, you will find more information on it at the end of the study.  In addition, you can download a report at the end of the study that provides an overview of the current state of research.  Please keep in mind that participation is only possible from the age of 14 onwards.  Thank you very much for your commitment!  Leonie Flacke, B. Sc.  PD Dr. Meinald Thielsch  Prof. Dr. Russell Haines  If you have questions or comments regarding the study, please send an e-mail at leonie.flacke@uni-muenster.de |
| --- |

**Page 2**

| [Introductory instructions:]  **Dear participant,**   prior to the start of the study you will receive several general comments regarding the completion of the study.  This study is about the reading and processing of medical information. This information will be prepared for you in short texts. With the reading, we ask you to answer a few questions. In total, this study includes three different tasks. For answering these questions, however, you do not need any previous knowledge. Additionally, you will be asked for your personal opinions after each task as well as at the end.  You can stop the study at any time and you can indicate at the end whether we may use your details for research purposes.  By clicking on “Proceed”, you declare that you are ready to participate in the study and that we may use your data.  Please do not use any auxiliary help (search engines etc.) during the study, as these would spoil the study’s results. We ask for your understanding that we will check in our study whether you used any auxiliary means, as the study loses meaningfulness otherwise. |
| --- |

**Page 3**

| [Demographics:]  First, please answer the following demographic questions. |
| --- |
|  |

**Page 4**

| [Screen size, keyboard needed]  For the correct presentation of the questions, please make sure to maximize your browser window and optimally switch to full-screen mode (Windows: F11, Mac: command + shift + F, in same this way you can also leave the mode), especially if you use a rather small screen (under 15"/ 38.1 cm screen diagonal).  You should work on this survey with a device that has a keyboard (i.e. not with a smartphone, tablet or the like), as you will partly work on tasks not with the mouse, but with the keyboard. |
| --- |

**Page 5**

| [Colorblindness Check:]  Please put the number next to each picture that you recognize. If you can’t recognize a number, please type “0.”  Are you colorblind? |
| --- |

**Page 6**

| Before the study starts, we ask you to answer the following questions regarding your current mood and motivation.  [Mood item]  Please work under the following conditions as you complete your tasks:  [Learning goal:] In the following, you will find a text about a medical issue. It is about aphasias. Please read this carefully. You have the **chance** to **learn** something **about aphasias**. Please use this chance and try to take in as much information as possible. After this, we will ask you a few questions that you can answer with the help of the text. Once you finish the study, you can win one of three gift cards worth €50, €25 or €15.    [Performance goal:] In the following, you will find a text about a medical issue. It is about aphasias. Please read this carefully. After this, we will test your reading comprehension using several questions. We will add up your points from all three tasks at the end of the study. The point score has an influence on the contest for the gift cards. The three participants **with the highest point score** by the end of the study receive a **gift card** worth €50 (1^st^ place), €25 (2^nd^ place) or €15 (3^rd^ place).  [Motivation pre item]  Are you ready?  We will now begin with the first task. For technical reasons, you will be redirected from this survey window to another platform as soon as you click on “Proceed”. |
| --- |

**Page 7 (from now on: Manipulation of aesthetics – high vs. low & Manipulation of goal orientation – learning vs. performance => 2 x 2 = 4 groups; group is non-varying over time)**

| [Task 1 (search-and-find):]  Broca’s aphasia  By aphasias we understand central, acquired dysfunctions of the language. The cognitive functions of aphasics are, however, not generally disturbed. Aphasias can be acquired by strokes or tumors, for example. Surgeries or injuries can also lead to the occurrence of aphasia. Aphasia is often a multimodal dysfunction, as it can also impair the listening or reading. Thus, aphasia can cause that these humans cannot read and/or write anymore, either, which we call alexia or agraphia, respectively. One form of aphasia is broca’s aphasia, which stems from the French neurologist Paul Broca. Broca aphasia concerns the language acquisition and is thus often described as motoric or also expressive aphasia. Patients complain about strong efforts while speaking. Their way of speaking is not fluent and stammering; articulation is bad. Additionally, they speak with little cadence. Patients speak very slowly and mainly use words of content. They have problems with the syntax and thus use an abrupt style while (spontaneously) speaking, which is called agrammatism, not to be confused with paragrammatism or dysgrammatism. Patients can usually only use one two three word-sentences; the formation of main and subordinate causes them great problems. The broca’s aphasics’ speech comprehension is, however, most extensively intact so that they only rarely show problems with word meaning. Nevertheless, we have to distinguish broca’s aphasia from pure speaking disorders like, for example, lisping, stuttering or mutism (partial or complete non-talking for a relatively long period, even though the linguistic development is mostly completed). By speaking disorders, we denote the inability to articulate speech sounds correctly and fluently. Contrary to the speech disorder, only motoric-articulatory skills are impaired; patients do not have any problems concerning the faculty of speech. A speech disorder can occur together with a speaking disorder.  Broca’s aphasia stems from a damage of the broca speech centre: it is located in the frontal lobe of the brain in the so-called areal 44 after Brodmann.  (Sources: Dijkstra, T. (1993). Einführung in die Psycholinguistik. Bern, Göttingen, Toronto, Seattle: Huber; http://flexikon.doccheck.com/de/Broca-Aphasie; https://de.wikipedia.org/wiki/Sprechst%C3%B6rung)  [Learning Goal:] Please answer the following questions. Use the chance to learn as much as possible about aphasias.  [Performance Goal:] Please answer the following questions. Keep in mind that the three best participants will win a gift card.   1. What is meant by agrammatism?   Solution: abrupt style/ no complete syntax / one to three word-sentences (1 Point)  2.) What do you call the incapability to read?  Solution: alexia (1 Point)  3.) In which Brodmann areal is broca’s aphasia located?  Solution: 44 (1 Point)  4.) How can aphasias be acquired?  Solution: stroke, tumor, surgery, injury (4 Points)  5.) What do most broca’s aphasics have no problems with?  Solution: with speech comprehension/ with word meaning (1 Points)  [Maximum: 8 points] |
| --- |

**Page 8**

| Post-task motivation, mood, stress |
| --- |

**Page 9**

| On the next page, you will find the second task. On this website, there is the possibility for you as the user to become active. You will find on this website the appeal to bring forward your own ideas.  [Motivation Pre2] |
| --- |

**Page 10**

| [Task 2 (creative)]  [Instructions above the embedded website:]  Here you can now see the web site with the appeal. Under it you will find the text field.  [Scenario on the website:] We need your help!  A person was taken to hospital with an unclear diagnosis. One important medical test is scheduled for the day after tomorrow. This person is very nervous. With which activities could you distract this person the day before the test? There are no medical limitations for the person.  [Learning goal:] You have got the **chance** with your ideas to make **a little joy** to someone having a hard time. For that reason, these ideas could be **helpful** for you later in your own everyday life. Please write down your ideas in bullet points in the text field below.  [Performance goal:] Be **better** than the other participants! This task will be scored afterwards. The **point score** you earn here has got an influence on the contest for **gift cards** at the end of the study. Please write down your ideas in bullet points in the text field below. |
| --- |

**Page 11**

| Post-task motivation, mood, stress |
| --- |

**Page 12**

| The third task follows on the next page. Because of technical reasons you cannot click on the various fields in the navigation bar anymore.  Please work on the following task:  [Learning goal:] In the following, you will find a new text about a medical issue. It is about another form of aphasia, the Wernicke’s Aphasia. Please read the text carefully and remember the text about Broca’s aphasia. After that, **you can try being a doctor**. Below you will find a speech example of an aphasics and please try to figure out which kind of aphasia it is. Altogether, you have the chance to get to know the everyday work at a hospital. Because it is a fictitious example for training, you do not need to be afraid of making a mistake.  [Performance goal:] In the following, you will find a new text about a medical issue. It is about another form of aphasia, the Wernicke’s Aphasia. Please read the text carefully and remember the text about Broca’s aphasia. After that, **you go further on the point hunt**. Below you will find a speech example of an aphasics and please try to figure out which kind of aphasia it is. Please think about the further consequences that can be caused in reality by a wrong decision. A right diagnosis is also beneficial here for your point account.  [Motivation Pre3] |
| --- |

**Page 13**

| Task 3 (transfer) [just a screenshot of the website; new text, but participants cannot click anything on the navigation bar]  [Instructions above the embedded website:]  Here you now see the website with the text about Wernicke-Aphasie. Under that you will find the speech example for this and a text field for your diagnosis  [On the website (screenshot)] Another known aphasia is the Wernicke’s aphasia. It goes back to the German neurologist Carl Wernicke. Patients with Wernicke’s aphasia still have got a good oral fluency but they often suffer from an excessive speaking production. Often, they develop an uninhibited oral fluency however the content is senseless. Moreover, having problems with finding words is typical for Wernicke aphasics. The speech comprehension and speech perception are immensely disordered. Wernicke’s aphasia is related to paragrammatism. Paragrammatism means the usage of long, complex sentences that are not finished correctly. In addition, Wernicke aphasics often use neologisms. Neologism means inventing new words that do not exist in the particular language. Patients with Wernicke’s aphasia often speak in a normal speed and rarely have problems articulating. The most common reason for an occurrence of Wernicke’s aphasia is a stroke. In the process of the stroke the Wernicke speech centre (brodmann areal 22) is damaged, which is important for speech comprehension.  (Source: Dijkstra, T. (1993). Einführung in die Psycholinguistik. Bern, Göttingen, Toronto, Seattle: Huber; http://flexikon.doccheck.com/de/Wernicke-Aphasie)  [Below the embedded website]  Speech example:  Examiner: „when you had a stroke, where were you at that time?“  Patient: “yes… me alone… alone alone… and far… flat… alone… Me me me… on-… only… and 14 days… I couldn’t anything… and wife J… yes my god… Miss J… help help… and pol… police… police and fire brigade… so wait…. I couldn’t any longer… so wait…. and that’s… St. Elisabethen hospital… that is good…”  [Learning Goal:] Now try being a doctor! What kind of aphasia is this speech example?  [Performance Goal:] Collect further points in order to win the gift card! What kind of aphasia is this speech example?  Solution: broca’s aphasia  (source: Dijkstra, T. (1993). Einführung in die Psycholinguistik. Bern, Göttingen, Toronto, Seattle: Huber) |
| --- |

**Page 14**

| Post-task motivation, mood, stress |
| --- |

**Page 15 (no manipulation anymore; separation of VisAWI + other control measures)**

| [Aesthetics:]  Here you see the design of the website again. Please evaluate the following statements about the design of the website. Answer as spontaneously as possible. Keep in mind that we are interested in your subjective evaluation, and that there are no right and wrong answers.   \| A screenshot of the website they had to deal with \| Show Design (the one they had to deal with) \| \| --- \| --- \| |
| --- | --- | --- |

**Page 16**

| Questionnaire about overall website impressions: ease of use, etc. |
| --- |

**Page 17**

| [Manipulation check:]  Please answer following questions.  [Goal orientation manipulation checks (two questions):]  Which of the following instructions has more priority in your opinion about individual learning growth?   \| In the following, you will find a text about a medical issue. It is about aphasias. Please read this carefully. You have the chance to learn something about aphasias. Please use this chance and try to take in as much information as possible. After this, we will ask you a few questions that you can answer with the help of the text. Once you finish the study, you can win one of three gift cards worth €50, €25 or €15. \| In the following, you will find a text about a medical issue. It is about aphasias. Please read this carefully. After this, we will test your reading comprehension using several questions. We will add up your points from all three tasks at the end of the study. The point score has an influence on the contest for the gift cards. The three participants with the highest point score by the end of the study receive a gift card worth €50 (1st place), €25 (2nd place) or €15 (3rd place). \| \| --- \| --- \|     [ ] [ ]  Which of the following instructions has more priority in your opinion about goal performance?   \| You have got the chance with your ideas to make a little joy to someone having a hard time. For that reason, these ideas could be helpful for you later in your own everyday life. Please write down your ideas in bullet points in the text field below. \| Be better than the other participants! This task will be scored afterwards. The point score you earn here has got an influence on the contest for gift cards at the end of the study. Please write down your ideas in bullet points in the text field below. \| \| --- \| --- \|   [ ] [ ]  [Aesthetics manipulation check:]  Which design did you like better?   \| Screenshot Design 1 \| Screenshot Design 2 \| \| --- \| --- \|   [ ] [ ] |
| --- | --- | --- | --- | --- | --- | --- |

**Page 18**

| Acknowledgement & explication  **Dear participants,**  thank you for your participation. This study intends to examine which influence the aesthetics of a user interface has on an individual’s performance. Because of that, it is possible that you experienced the design of the study as perhaps very pretty or as very unattractive. The website was especially created for this study. It is not a real website. Moreover, there were two different instructions that were meant to manipulate the goal orientation. In one group, the learning potential was stressed (Learning Goal), in the other group the result (Performance Goal). In the performance goal group, the instructions might have sounded strict and direct; maybe you felt pressured because of that. Those instructions should not bother you, instead they were necessary for the experimental manipulation. Altogether, you were assigned to one of four possible groups in this study (Pretty/Learning; Pretty/Performance; Unattractive/Learning; Unattractive/Performance). In fairness, every group naturally gets the same number of gift cards. Per group, there are three gift cards in total worth 50€, 25€ or 15€ to win.  Do you have got any remarks? (If not, write simply “No”)   \|  \| \| --- \|   The study is almost finished. Please click “Proceed”! |
| --- | --- |

**Page 19**

| Permission to use their anonymous data. |
| --- |

**Page 20**

| Results  [Contest participation: Those that wish to participate instructed to enter email and acknowledge that points scored will be associated with email address. Those not participating instructed to simply close the window.]  [Further information about aesthetics and performance: Opportunity to download a pdf, notification that results of this study will be available on PsyWeb at the end of the year.] |
| --- |
