# Supplementary material for: Experimental investigation on the effects of website aesthetics on user performance in different virtual tasks
Source: PeerJ. 2019 Feb 22;7:e6516. doi: 10.7717/peerj.6516 (PMC6388663; doi:10.7717/peerj.6516)
Supplement: Supplemental Information 1 — Includes original instructions and items, their translation into English, raw data (without demographics), and a coding scheme for the open answers. [file peerj-07-6516-s001.zip › Data_Package/Instructions_German_Original.docx]

**Instructions**

**Page 1**

| [Welcome & introduction:]  **Studie zum Lesen und Verarbeiten von medizinischen Informationen**  **Liebe Teilnehmerin, lieber Teilnehmer,**  vielen Dank, dass Sie sich entschlossen haben, bei dieser Studie mitzuwirken. Wir führen diese Studie im Rahmen einer Forschungsarbeit im Fach Psychologie an der Westfälischen Wilhelms-Universität Münster durch. In dieser Studie werden Sie gebeten, sich mit medizinischen Themen auseinander zu setzen. Sie benötigen jedoch keinerlei Vorwissen. Alle in der Studie erworbenen Daten werden selbstverständlich nicht auf individueller Ebene, sondern zu Forschungszwecken gemittelt ausgewertet sowie anonymisiert erfasst.  Die Studie dauert circa 25 - 30 Minuten.  Im Anschluss an die Studie haben Sie die Chance, einen von drei Buchgutscheinen im Wert von 50 €, 25 € oder 15 € zu gewinnen. Wenn Sie an der Verlosung teilnehmen möchten, finden Sie am Ende der Studie weitere Informationen hierzu.  Darüber hinaus können Sie sich am Ende der Studie einen Bericht herunterladen, der einen Überblick zum bisherigen Forschungsstand gibt.  Bitte beachten Sie, dass eine Teilnahme erst ab einem Alter von 14 Jahren möglich ist.  Vielen Dank für Ihr Engagement!  Leonie Flacke, B. Sc.  PD Dr. Meinald Thielsch  Prof. Dr. Russell Haines  Bei Fragen oder Anmerkungen zur Studie wenden Sie sich bitte per Mail an leonie.flacke@uni-muenster.de |
| --- |

**Page 2**

| [Introductory instructions:]  **Liebe Teilnehmerin, lieber Teilnehmer,**  vor Beginn der Studie erhalten Sie einige allgemeine Hinweise zur Durchführung dieser Studie.  In dieser Studie geht es um das Lesen und Verarbeiten von medizinischen Informationen. Diese Informationen wurden für Sie in kurzen Texten aufbereitet. Neben dem Lesen bitten wir Sie, einige Fragen zu beantworten. Insgesamt beinhaltet diese Studie drei verschiedene Aufgaben. Zur Beantwortung der Fragen benötigen Sie jedoch keinerlei Vorwissen. Zusätzlich werden Sie nach jeder Aufgabe sowie am Ende um persönliche Einschätzungen gebeten.  Sie können die Studie jederzeit abbrechen und am Ende bestimmen, ob wir Ihre Angaben zu Forschungszwecken verwenden dürfen.  Mit dem Klick auf "Weiter" erklären Sie, dass Sie bereit sind, an der Studie teilzunehmen und dass wir Ihre Daten verwenden dürfen.  Bitte benutzen Sie während der Studie keine Hilfsmittel (Suchmaschinen etc.), da diese die Studienergebnisse verfälschen würden. Wir bitten um Ihr Verständnis, dass wir in unserer Studie überprüfen werden, ob Sie Hilfsmittel verwendet haben, da die Studie sonst an Aussagekraft verliert. |
| --- |

**Page 3**

| [Demographics:]  Bitte beantworten Sie zunächst die folgenden demografischen Fragen. |
| --- |
|  |

**Page 4**

| [Screen size, keyboard needed]  Zur korrekten Darstellung der Fragen achten Sie bitte darauf, Ihr Browserfenster zu maximieren und optimalerweise auf Vollbildmodus umzustellen (Windows: F11, Mac: command + Shift + F, genauso können Sie den Modus auch wieder verlassen), vor allem wenn Sie einen eher kleinen Bildschirm verwenden (unter 15"/ 38,1 cm Bildschirmdiagonale).  Sie sollten diese Umfrage mit einem Gerät bearbeiten, das eine Tastatur hat (also nicht mit einem Smartphone, Tablet o.ä.), da Sie die Aufgaben teilweise nicht mit der Maus, sondern mit der Tastatur bearbeiten werden. |
| --- |

**Page 5**

| [Colorblindness Check:]  Bitte geben Sie neben jedem Bild die Zahl an, die Sie erkennen können. Wenn Sie keine Zahl erkennen können, tippen Sie bitte „0“ ein.  Leiden Sie unter einer Farbfehlsichtigkeit? |
| --- |

**Page 6**

| Bevor es richtig losgeht, bitten wir Sie, die folgenden Fragen zu Ihrer aktuellen Stimmung und Motivation zu beantworten.  [Mood item]  Bitte bearbeiten Sie gleich folgende Aufgabenstellung:  [Learning goal:] Im Folgenden erwartet Sie ein Text zu einem medizinischen Thema. Es geht um Aphasien. Bitte lesen Sie sich diesen aufmerksam durch. Sie haben die **Chance**, etwas **über Aphasien zu lernen**. Nutzen Sie diese Chance und versuchen Sie, so viele Informationen wie möglich für Sie persönlich mitzunehmen. Im Anschluss stellen wir Ihnen ein paar Fragen, die Sie mit Hilfe des Textes beantworten können. Wenn Sie die Studie beenden, können Sie einen von drei Gutscheinen in Höhe von 50 €, 25 € oder 15 € gewinnen.    [Performance goal:] Im Folgenden erwartet Sie ein Text zu einem medizinischen Thema. Es geht um Aphasien. Bitte lesen Sie diesen Text aufmerksam durch. Im Anschluss testen wir Ihr Textverständnis mithilfe einiger Fragen. Wir addieren Ihre Punkte aus allen drei Aufgaben am Ende der Studie. Diese Punktzahl hat einen Einfluss auf die Verlosung der Gutscheine. Die drei Teilnehmer **mit der höchsten Punktzahl** am Ende der Studie erhalten einen **Gutschein** in Höhe von 50 € (1. Platz), 25 € (2. Platz) oder 15 € (3. Platz).  [Motivation pre item]  Sind Sie bereit?  Wir beginnen nun mit der ersten Aufgabe. Dafür werden Sie aus technischen Gründen von diesem Umfragefenster auf eine weitere Plattform umgeleitet, sobald Sie auf „Weiter“ klicken. |
| --- |

**Page 7 (from now on: Manipulation of aesthetics – high vs. low & Manipulation of goal orientation – learning vs. performance => 2 x 2 = 4 groups; group is non-varying over time)**

| [Task 1 (search-and-find):]  Hier sehen Sie nun die Website mit dem Text über Broca-Aphasie. Darunter finden Sie die dazugehörigen Fragen.  Broca-Aphasie  Unter Aphasien versteht man zentrale, erworbene Störungen der Sprache. Die kognitiven Funktionen von Aphasikern sind jedoch nicht generell gestört. Erworben werden können Aphasien beispielsweise durch Schlaganfälle oder Tumore. Zum Auftreten einer Aphasie können auch Operationen oder Verletzungen führen. Aphasie ist oft eine multimodale Störung, da sie auch das Zuhören oder Lesen beeinträchtigen kann. Somit kann Aphasie dazu führen, dass die Menschen auch nicht mehr lesen und/oder nicht mehr schreiben können, was man als Alexie beziehungsweise Agraphie bezeichnet. Eine Form der Aphasie ist die Broca-Aphasie, welche auf den französischen Neurologen Paul Broca zurückgeht. Eine Broca-Aphasie betrifft die Sprachbildung und wird daher häufig als motorische oder auch expressive Aphasie bezeichnet. Patienten klagen über starke Anstrengungen beim Sprechen. Ihre Sprechweise ist nicht flüssig und stammelnd; die Artikulation ist schlecht. Sie sprechen zudem mit geringer Sprachmelodie. Die Patienten sprechen sehr langsam und verwenden hauptsächlich Inhaltswörter. Sie haben Probleme mit der Syntax und verwenden somit einen Telegrammstil beim (spontanen) Sprechen, was man Agrammatismus, nicht zu verwechseln mit Paragrammatismus oder Dysgrammatismus, nennt. Die Patienten können zumeist nur Ein- bis Dreiwortsätze verwenden; die Bildung von Haupt- und Nebensätzen bereitet ihnen sehr große Probleme. Das Sprachverständnis von Broca-Aphasikern ist jedoch weitestgehend intakt, sodass sie nur selten Probleme mit der Wortbedeutung aufweisen. Allerdings muss man die Broca-Aphasie von reinen Sprechstörungen wie beispielsweise Lispeln, Stottern oder Mutismus (teilweises oder vollständiges Nichtsprechen über einen relativ langen Zeitraum hinweg, obwohl die Sprachentwicklung weitgehend abgeschlossen ist) abgrenzen. Als Sprechstörung bezeichnet man die Unfähigkeit, Sprachlaute korrekt und fließend zu artikulieren. Im Gegensatz zur [Sprachstörung](https://de.wikipedia.org/wiki/Sprachst%C3%B6rung) sind hier nur die motorisch-artikulatorischen Fertigkeiten beeinträchtigt; Patienten haben keine Probleme mit dem Sprachvermögen. Eine Sprachstörung kann auch gemeinsam mit einer Sprechstörung auftreten.  Die Broca-Aphasie ist auf eine Schädigung des Broca-Sprachzentrums zurückzuführen: Dieses befindet sich im Stirnlappen des Gehirns in dem sogenannten Areal 44 nach Brodmann.  (Quellen: Dijkstra, T. (1993). Einführung in die Psycholinguistik. Bern, Göttingen, Toronto, Seattle: Huber; http://flexikon.doccheck.com/de/Broca-Aphasie; https://de.wikipedia.org/wiki/Sprechst%C3%B6rung)  [Learning Goal:] Bitte beantworten Sie nun die folgenden Fragen. Nutzen Sie die Chance, möglichst viel über Aphasien zu lernen.  [Performance Goal:] Bitte beantworten Sie nun die folgenden Fragen. Denken Sie daran, dass die besten drei Teilnehmer einen Gutschein gewinnen.  1.) Was versteht man unter Agrammatismus?  Lösung/solution: Telegrammstil / keine vollständige Syntax / Ein- bis Dreiwortsätze (1 Point)  2.) Wie bezeichnet man das Unvermögen zu lesen?  Lösung/solution: Alexie (1 Point)  3.) Welches Brodmann-Areal ist bei der Broca-Aphasie betroffen?  Lösung/solution: 44 (1 Point)  4.) Wie können Aphasien erworben werden?  Lösung/solution: Schlaganfall, Tumor, Operation, Verletzung (4 Points)  5.) Womit haben die meisten Broca-Aphasiker keine Probleme?  Lösung/solution: mit dem Sprachverständnis/ mit der Wortbedeutung (1 Point)  [Insgesamt 8 Punkte möglich] |
| --- |

**Page 8**

| Post-task motivation, mood, stress |
| --- |

**Page 9**

| Auf der nächsten Seite folgt die zweite Aufgabe. Auf dieser Website gibt es die Möglichkeit, als User selbst aktiv zu werden. Sie finden auf der Website einen Appell, eigene Ideen hervorzubringen.  [Motivation Pre2] |
| --- |

**Page 10**

| [Task 2 (creative)]  [Instructions above the embedded website:]  Hier sehen Sie nun die Website mit dem Appell. Darunter finden Sie das Textfeld.  [Scenario on the website:] Wir brauchen Ihre Hilfe!  Eine Person wurde mit einer unklaren Diagnose ins Krankenhaus eingeliefert. Eine wichtige Untersuchung wurde für übermorgen angesetzt. Diese Person ist sehr nervös. Mit welchen Aktivitäten könnten Sie diese Person am Tag vor der Untersuchung ablenken? Es bestehen keine medizinischen Einschränkungen.  [Learning goal:] Sie erhalten nun die **Chance**, einem Menschen durch Ihre Ideen eine **kleine Freude** in einer für ihn schweren Zeit zu machen. Zudem können Ihnen diese Ideen später einmal im Alltag **behilflich** sein. Schreiben Sie Ihre Ideen bitte stichwortartig in das unten stehende Textfeld.  Performance goal: Seien Sie **besser** als die anderen Teilnehmer! Diese Aufgabe wird im Anschluss ausgewertet. Ihre hier erreichte **Punktzahl** hat ebenfalls einen Einfluss auf die Verlosung der **Gutscheine** am Ende der Studie. Schreiben Sie Ihre Ideen bitte stichwortartig in das unten stehende Textfeld. |
| --- |

**Page 11**

| Post-task motivation, mood, stress |
| --- |

**Page 12**

| Auf der nächsten Seite folgt die dritte Aufgabe. Aus technischen Gründen können Sie die verschiedenen Felder in der Navigationsleiste nun nicht mehr anklicken.    Bitte bearbeiten Sie gleich folgende Aufgabenstellung:  [Learning goal:] Im Folgenden erwartet Sie erneut ein Text zu einem medizinischen Thema. Es geht um eine weitere Form der Aphasie, die Wernicke-Aphasie. Bitte lesen Sie diesen Text aufmerksam durch und erinnern sich zudem an den Text zur Broca-Aphasie. Dann **dürfen Sie sich einmal** **als Arzt ausprobieren**. Unten erhalten Sie ein Sprachbeispiel von einem Aphasiker und versuchen bitte herauszufinden, um welche Art von Aphasie es sich handelt. Insgesamt erhalten Sie die Chance, die tagtägliche Arbeit im Krankenhaus kennenzulernen. Da es sich um ein fiktives Beispiel zum Üben handelt, brauchen Sie keine Angst haben, einen Fehler zu machen.  [Performance goal:] Im Folgenden erwartet Sie erneut ein Text zu einem medizinischen Thema. Es geht um eine weitere Form der Aphasie, die Wernicke-Aphasie. Bitte lesen Sie diesen Text aufmerksam durch und erinnern sich zudem an den Text zur Broca-Aphasie. Dann **gehen Sie weiter auf Punktejagd**. Unten erhalten Sie ein Sprachbeispiel von einem Aphasiker und finden bitte heraus, um welche Art von Aphasie es sich handelt. Denken Sie bitte an die weitreichenden Konsequenzen, die durch eine falsche Entscheidung in der Realität ausgelöst werden könnten. Auch hier wirkt sich eine richtige Diagnose natürlich positiv auf Ihr Punktekonto aus.  [Motivation Pre3] |
| --- |

**Page 13**

| Task 3 (transfer) [just a screenshot of the website; new text, but participants cannot click anything on the navigation bar]  [Instructions above the embedded website:]  Hier sehen Sie nun die Website mit dem Text über Wernicke-Aphasie. Darunter finden Sie das dazugehörige Sprachbeispiel und ein Textfeld für Ihre Diagnose  [On the website (screenshot)] Eine weitere bekannte Aphasie ist die Wernicke-Aphasie. Sie geht zurück auf den deutschen Neurologen Carl Wernicke. Bei Patienten mit Wernicke-Aphasie ist der Redefluss gut erhalten, allerdings leiden sie oft sogar unter einer überschießenden Sprachproduktion. Es entsteht so häufig ein ungehemmter Redefluss mit jedoch sinnlosem Inhalt. Zudem sind Wortfindungsprobleme typisch für Wernicke-Aphasiker. Das Sprachverständnis sowie die Sprachwahrnehmung von diesen Patienten sind erheblich gestört. Mit der Wernicke-Aphasie wird zudem der Paragrammatismus verbunden. Unter Paragrammatismus versteht man den Gebrauch von langen, komplexen Sätzen, welche aber oft nicht korrekt beendet werden. Wernicke-Aphasiker verwenden außerdem häufig Neologismen. Als Neologismen bezeichnet man neu erfundene Wörter, die es in der jeweiligen Sprache so nicht gibt. Patienten mit Wernicke-Aphasie sprechen zumeist in normaler Geschwindigkeit und haben auch kaum Probleme, sich zu artikulieren. Die häufigste Ursache für das Auftreten der Wernicke-Aphasie ist der Schlaganfall. Im Zuge dessen kommt es zu Verletzungen des Wernicke-Sprachzentrums (Brodmann Areal 22), welches für das Sprachverständnis wichtig ist.  (Quellen: Dijkstra, T. (1993). Einführung in die Psycholinguistik. Bern, Göttingen, Toronto, Seattle: Huber; http://flexikon.doccheck.com/de/Wernicke-Aphasie)  [Below the embedded website]  Sprachbeispiel:  Untersucher: „Als Sie damals einen Schlaganfall bekamen, wo waren Sie damals?“  Patient: „ja…ich allein…allein allein…und weit…Wohnung…allein…ich ich ich…le…ledig…und 14 Tage…ich konnt nichts…und Frau J….ja meine Güte…Frau J…Hilfe Hilfe…und Pol…Polizei…Polizei und Feuerwehr…so warte…ich konnt nicht mehr…so warte…und des is…St. Elisabethen Krankenhaus…des is gut…“  [Learning Goal:] Probieren Sie sich nun einmal als Arzt aus! Für welche Art von Aphasie spricht dieses Sprachbeispiel?  [Performance Goal:] Sammeln Sie weitere Punkte, um den Gutschein zu gewinnen! Für welche Art von Aphasie spricht dieses Sprachbeispiel?  Lösung/solution: Broca-Aphasie  (entnommen aus: Dijkstra, T. (1993). Einführung in die Psycholinguistik. Bern, Göttingen, Toronto, Seattle: Huber) |
| --- |

**Page 14**

| Post-task motivation, mood, stress |
| --- |

**Page 15 (no manipulation anymore; separation of VisAWI + other control measures)**

| Aesthetics:  Hier sehen Sie erneut das Design der Website. Bitte beurteilen Sie die folgenden Aussagen in Bezug auf das Design der Website. Antworten Sie bitte möglichst spontan. Beachten Sie bitte, dass uns Ihre subjektive Einschätzung interessiert und es hierbei keine richtigen und falschen Antworten gibt.   \| A screenshot of the website they had to deal with \| \| --- \|   **VisAWI questions** |
| --- | --- |

**Page 16**

| Questionnaire about overall website impressions: ease of use, etc. |
| --- |

**Page 17**

| [Manipulation check:]  Bitte beantworten Sie die folgenden Fragen.  [Goal orientation manipulation checks (two questions):]  Bei welcher Anweisung steht Ihrer Meinung nach der individuelle Lernzuwachs stärker im Vordergrund?   \| Im Folgenden erwartet Sie ein Text zu einem medizinischen Thema. Es geht um Aphasien. Bitte lesen Sie sich diesen aufmerksam durch. Sie haben die Chance, etwas über Aphasien zu lernen. Nutzen Sie diese Chance und versuchen Sie, so viele Informationen wie möglich für Sie persönlich mitzunehmen. Im Anschluss stellen wir Ihnen ein paar Fragen, die Sie mit Hilfe des Textes beantworten können. Wenn Sie die Studie beenden, können Sie einen von drei Gutscheinen in Höhe von 50 €, 25 € oder 15 € gewinnen. \| Im Folgenden erwartet Sie ein Text zu einem medizinischen Thema. Es geht um Aphasien. Bitte lesen Sie diesen Text aufmerksam durch. Im Anschluss testen wir Ihr Textverständnis mithilfe einiger Fragen. Wir addieren Ihre Punkte aus allen drei Aufgaben am Ende der Studie. Diese Punktzahl hat einen Einfluss auf die Verlosung der Gutscheine. Die drei Teilnehmer mit der höchsten Punktzahl am Ende der Studie erhalten einen Gutschein in Höhe von 50 € (1. Platz), 25 € (2. Platz) oder 15 € (3. Platz). \| \| --- \| --- \|   [ ] [ ]  Bei welcher Anweisung steht Ihrer Meinung nach die erzielte Leistung stärker im Vordergrund?   \| Sie erhalten nun die Chance, einem Menschen durch Ihre Ideen eine kleine Freude in einer für ihn schweren Zeit zu machen. Zudem können Ihnen diese Ideen später einmal im Alltag behilflich sein. Schreiben Sie Ihre Ideen bitte in das unten stehende Textfeld. \| Seien Sie besser als die anderen Teilnehmer! Diese Aufgabe wird im Anschluss ausgewertet. Ihre hier erreichte Punktzahl hat ebenfalls einen Einfluss auf die Verlosung der Gutscheine am Ende der Studie. Schreiben Sie Ihre Ideen bitte in das unten stehende Textfeld. \| \| --- \| --- \|   [ ] [ ]  [Aesthetics manipulation check:]  Welches Design finden Sie schöner?   \| Screenshot Design 1 \| Screenshot Design 2 \| \| --- \| --- \|   [ ] [ ] |
| --- | --- | --- | --- | --- | --- | --- |

**Page 18**

| Acknowledgement & explication  **Liebe Teilnehmerin, lieber Teilnehmer,**  vielen Dank für Ihre Teilnahme. Mit dieser Studie soll untersucht werden, welchen Einfluss die Ästhetik einer Benutzeroberfläche auf die individuelle Leistung hat. Deshalb kann es sein, dass Sie das Design der Studie vielleicht als sehr schön oder auch als besonders unschön empfunden haben. Die Website wurde eigens für diese Studie kreiert. Es handelt sich also nicht um eine reale Website. Außerdem gab es zwei verschiedene Anweisungen, mit der die Zielorientierung manipuliert werden sollte. In der einen Gruppe wurde das Lernpotenzial betont (Learning Goal), bei der anderen das Ergebnis (Performance Goal). In der Performance-Goal-Gruppe klangen die Anweisungen daher für Sie eventuell ziemlich streng und direkt; vielleicht haben Sie sich deshalb etwas unter Druck gesetzt gefühlt. Die Anweisungen sollten Sie folglich nicht schikanieren, sondern dienten lediglich der experimentellen Manipulation. Insgesamt gehörten Sie in dieser Studie zu einer von vier möglichen Gruppen (Schön/Learning; Schön/Performance; Unschön/Learning; Unschön/Performance). Der Fairness halber wird selbstverständlich in jeder Gruppe die gleiche Anzahl an Gutscheinen ausgegeben. Pro Gruppe gibt es insgesamt drei Gutscheine im Wert von entweder 50 €, 25 € oder 15 € zu gewinnen.  Haben Sie Anmerkungen? (Wenn nicht, schreiben Sie einfach „Nein“)   \|  \| \| --- \|   Die Studie ist fast beendet. Bitte klicken Sie auf „Weiter“! |
| --- | --- |

**Page 19**

| Permission to use their anonymous data. |
| --- |

**Page 20**

| Results  [Contest participation: Those that wish to participate instructed to enter email and acknowledge that points scored will be associated with email address. Those not participating instructed to simply close the window.]  [Further information about aesthetics and performance: Opportunity to download a pdf, notification that results of this study will be available on PsyWeb at the end of the year.] |
| --- |
